# A free ‘app’ for plastic surgery: the smartphone spirit level

**DOI:** 10.1308/003588412X13373405387096k

**Published:** 2012-05

**Authors:** A Souéid, A Khanna

**Affiliations:** Sandwell and West Birmingham Hospitals NHS Trust,UK

## BACKGROUND

Symmetry is one of the criteria of achieving a good aesthetic result. Realising this can be difficult. Attempts have been made to try and measure this symmetry using advanced and complex methods.^1^ For most surgeons on a daily basis the only practical way to measure symmetry is to use a simple measuring tool such as a measuring tape. However, this method is not very reliable as it depends on the patient being in a totally straight position, the measuring tape being accurate to 1mm and the tape not moving when comparing both sides.

## TECHNIQUE

We describe the use of an iPhone® spirit level application (FreeSpirit; developer Claire Holmes), downloaded for free from iTunes®, that is helpful for this purpose. There are also other spirit level apps that can be downloaded for free on the iPhone® or Android™ phones.

**Figure 1:**
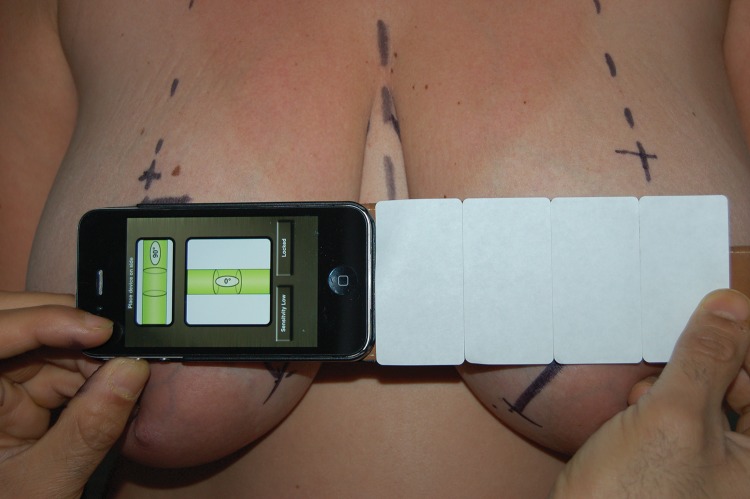
The application in use in the assessment of a patient undergoing breast surgery

By placing the spirit level in a horizontal position and centring the spirit point, the surgeon can check whether two corresponding body parts are on the same horizontal plane. The senior author finds this particularly useful when comparing inframammary folds or nipple positions in breast aesthetic and reconstructive surgery (Fig 1). Another application is in eye or eyebrow surgery to determine whether the eyebrows, eyelids or pupils are identical in their position on both sides.

## DISCUSSION

By sharing this technique, we hope to enable readers to add another tool to their armamentarium and aid them in trying to perfect their results.

## References

[1] Kawale M, Lee J, Leung SY*et al* 3D symmetry measure invariant to subject pose during image acquisition. Breast Cancer 2011; 5: 131–142 21792310 10.4137/BCBCR.S7140 PMC3140267

